# A five domains assessment of sow welfare in a novel free farrowing system

**DOI:** 10.3389/fvets.2024.1339947

**Published:** 2024-08-12

**Authors:** Kate Plush, David Lines, Lauren Staveley, Darryl D’Souza, Robert van Barneveld

**Affiliations:** SunPork Group, Brisbane, QLD, Australia

**Keywords:** sow, welfare, farrowing, lactation, piglet, Maternity Ring

## Abstract

The Maternity Ring was developed as a free farrowing alternative to crates that preserved space whilst providing the sow with unrestricted movement. This experiment aimed to apply the Five Domains model to assess sow welfare in the Maternity Ring in comparison with the farrowing crate. Eighty-eight sows were housed in a farrowing crate (FC) and 83 in a Maternity Ring (MR), and measures collected focussed on nutrition, environment, health, behaviour, and mental state outcomes. MR sows consumed less feed than FC sows (total feed intake: 93.8 ± 3.06 kg vs. 111.2 ± 3.13 kg; *p* < 0.001) but had reduced P2 backfat loss during lactation (0.0 ± 0.11 vs. 1.2 ± 0.11, *p* < 0.001). Fewer frustrated and pain-related behaviours during farrowing were observed in MR sows (bar biting: FC 3.3 ± 2.12 vs. MR 0.5 ± 0.29 events, *p* = 0.038, and back leg forward: FC 227 ± 50.7 vs. MR 127 ± 26.4 events, *p* = 0.019), and a decreased proportion of MR sows had facial injuries after farrowing (10% CI [5, 20] vs. 67% CI [47, 95], *p* < 0.001). More FC sows had udder damage at weaning (70% CI [48, 97] vs. 10% CI [6, 24], *p* < 0.001), and their piglets were medicated more frequently when compared to those in MR (51% CI [40, 61] vs. 30% [21, 41], *p* = 0.008). MR sows tended to have a higher reaction score to piglet processing (MR 2.0 ± 0.38 vs. FC 1.2 ± 0.27, *p* = 0.094) and had more contact with piglets once the procedure was complete than FC sows (13.5 ± 2.55 vs. 6.9 ± 1.26 events, respectively, *p* = 0.016). Whilst there was no difference in anticipation of a feeding event (*p* > 0.05), MR sows displayed a reduced startle response to an aversive noise stimulus at day 18 (FC 2.8 ± 0.35, MR 0.7 ± 0.16, *p* < 0.001). Using the Five Domains framework, sows housed in the MR during farrowing and lactation have improved welfare than those in FC and can be thought of as being in a positive affective state.

## 1 Introduction

The farrowing crate was designed to improve piglet survival and provide a safer environment for the stockperson, with the design also allowing farmers to maximise spatial requirements per sow and litter and the labour required to maintain hygiene (1). Whilst the farrowing crate is successful at protecting the piglet from injury and mortality (2) and increasing stockperson safety (3), there is evidence of a welfare cost associated with this system on the sow. There are two periods when the confinement of a sow within a farrowing crate would impact welfare: prior to farrowing when the sow has an intrinsic need to build a nest, and as lactation progresses when the sow begins to wean her litter.

Nesting behaviour is internally motivated by prepartum hormonal changes (4) and is terminated by sufficient external feedback from the nest site to confirm that the nest has been completed (5). When confined within a barren environment, sows will perform nesting behaviours by nosing, biting, and pawing crate fixtures, with these vacuum activities becoming stereotypic in nature. Bar biting has been suggested as an indicator of impaired welfare associated with confinement (6) and is believed to occur in response to the sow’s inability to satisfactorily nest in a crate (7). As lactation progresses, the restriction of movement under crated conditions prevents the sow from avoiding piglet attempts to feed resulting in painful udder sores and damage (8). Studies have consistently found a reduction in skin lesions on the udder of sows in farrowing pens when compared to crates (9–11). A recent analysis of nursing behaviour has identified that sows in pens are more successful at terminating feeding bouts as lactation progresses (11), closer aligning with how weaning occurs in the wild. Therefore, the use of farrowing pens can be assumed to be advantageous to sow welfare as they promote two natural behaviours in nest building (in the presence of enrichment) and sow-controlled weaning.

Free farrowing, as opposed to temporary crating, is the only practical option that provides an opportunity for sow welfare improvements both during nesting and in later lactation (12), but widespread adoption of such systems is largely absent. Baxter et al. (13) recently summarised the competing needs of pen system users (being pigs, farmers, and external stakeholders) and identified that ‘the pivotal starting point for those investing in new systems is the spatial footprint per sow place’. Therefore, the current predicament is how to provide enough space for the sow to have adequate movement before and during farrowing, and throughout lactation to improve welfare, whilst preserving space to limit negative financial impacts.

Traditionally, assessment of animal welfare has been based on the Five Freedoms (14) as a means of minimising the negative experiences of an animal, generally resulting in, at best, a neutral mental state. The aim of the Five Domains model (15) is to allow for positive experiences and create ‘a life worth living’ (16). The Five Domains framework investigates behaviour, nutrition, environment, and health and how these factors impact the animal’s mental state. Whilst the assessment of mental state in animals is controversial, reactions to perceived negative and positive events have been suggested as determinants of affective state (17). The defence cascade is the response of an animal to sudden, unexpected stimuli and involves initial detection and immediate response (startle) to a stimulus such as an unexpected noise (18–20), monitoring and evaluation of the stimulus accompanied by freezing/immobility (21, 22). The final response is either defensive/escape behaviour or more commonly the resumption of previous activities. Generally, animals with a more negative affect show attenuated startle and prolonged freeze behaviour in such a test (23). Anticipatory activity is defined as the behaviours that occur in the lead-up to a known positive event and is reflective of the emotional state of the animal (24). Stimuli that elicit anticipatory behavioural patterns are achieved through the repeated association with biologically relevant events and can be categorised as incentive stimuli (25, 26). The rewarding value of such stimuli is dependent on the current internal state of the animal, with a positive welfare state resulting in higher levels of anticipation of an incentivised event (27). To date, investigations into free farrowing that specifically apply the Five Domains model of animal welfare assessment are lacking. This investigation aimed to use the Five Domains model to assess the welfare of sows in a novel free farrowing design that preserves current sow space requirements, known as the Maternity Ring, in comparison with those housed in a farrowing crate. We hypothesised that the Maternity Ring would better meet the behavioural requirements of a sow during farrowing and lactation resulting in a more positive affective state.

## 2 Materials and methods

This experiment was conducted in accordance with the Australian Code of Practice for the Care and Use of Animals for Scientific Purposes (28) with approval from the Primary Industries and Regions South Australia (PIRSA) Animal Ethics Committee (Project number: 21/21).

### 2.1 Animal management

The experiment was conducted on a breeder unit with experimental sows (Camborough 42, PIC Australia, Grong Grong, NSW, Australia) mated in July and September 2021 and farrowed over two replicates in November (maximum average daily temperature 25.0 ± 5.9°C; minimum average daily temperature 10.7 °C± 4.4°C) and January (maximum average daily temperature 30.8 ± 4.7°C; minimum average daily temperature 16.5°C ± 4.6°C). One hundred and seventy-one sows (parity 2.6 ± 0.12) were observed from entry to the farrowing house until weaning. Sows were moved into the farrowing house 4.8 ± 0.21 days prior to farrowing. Farrowing sheds were cross-ventilated, with a temperature (>28°C) activated dripper system above every farrowing space to allow for evaporative cooling. Farrowing sheds were artificially lit from 0500 to 2,100. The standard farrowing space was 1.8 × 2.4 m (4.32m^2^), with fully slatted plastic flooring, a creep area, heated via lamp or mat for piglets, and an *ad libitum* feeder (Crystal Spring Hog Equipment, Ste. Agathe, Canada), two water nipples for the sow and one for the piglets. Sows were delivered 2 kg of a pre-farrow diet (12.75 MJ digestible energy (DW)/kg, 0.05 standardised ileal digestible (SID) lysine/MJ DE) at 07:00 and 15:00 prior to farrowing, with *ad libitum* access to a standard lactation diet (13.9 MJ digestible energy (DW)/kg, 0.6 standardised ileal digestible (SID) lysine/MJ DE) after farrowing. All sows were provided with a hessian bag (43 × 75 cm, Daish Irrigation and Fodder, Murray Bridge, SA, Australia) upon entry to the farrowing house for nesting. At approximately 24 h post-farrowing, piglets were cross-fostered where necessary within treatment. On day 2, piglets were tail docked using cauterisation, administered an iron injection (1 mL Feron 200 + B12 (200 mg/mL iron as iron dextran, 40 μg/mL cyanocobalamin), Elanco Australasia Pty Ltd., Macquarie Park, NSW, Australia), and oral coccidiostat (1 mL Baycox Piglet Coccidiocide (50 mg/mL Toltrazuril), Elanco Australasia Pty Ltd., Macquarie Park, NSW, Australia). Sow and piglets were weaned at 23.7 ± 0.23 days of lactation.

### 2.2 Housing treatment

**Figure 1:**
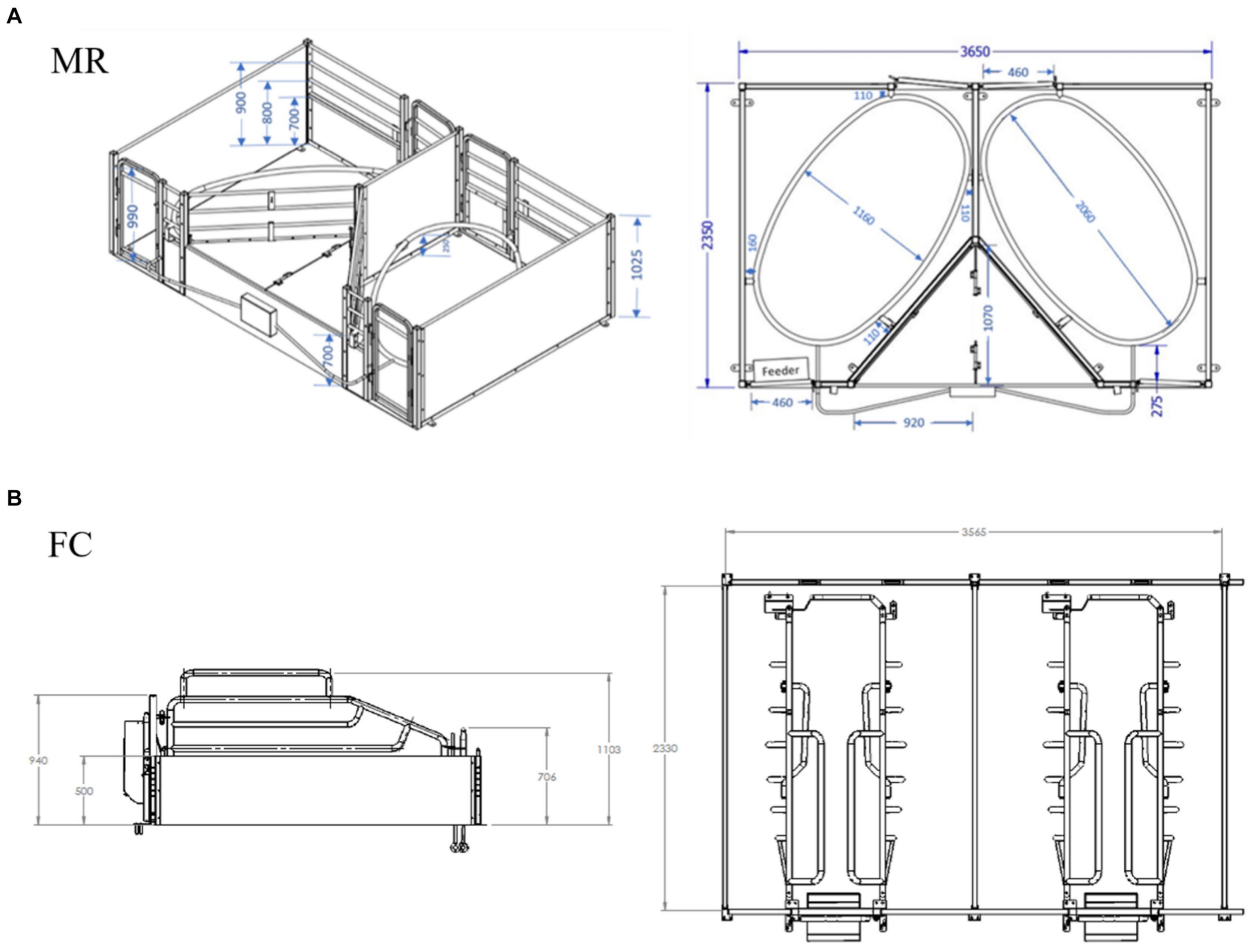
Dimensions of key design features of the **(A)** Maternity Ring (MR) and **(B)** farrowing crate (FC).

Sows were allocated to a standard farrowing crate (n = 88; FC) or a Maternity Ring (n = 83; MR) based on even parity distribution (Figure 1). The FC (Stockyard Industries, North Bendigo, VIC, Australia) was 1,783 mm in width by 2,330 mm in length, with a triangular creep area, measuring 840 mm deep and 820 mm wide. A back gate opposite the front gate provided two entry points to the pen. The crate was installed on polygrate plastic slatted flooring (Stockyard Industries, North Bendigo, VIC AU), within the pen parallel to the external dividers and measured 720 mm in width and 2,330 mm in length. The triangular piglet creep area (0.34 m^2^) was heated and lit via lamp (Vaucluse &APS Livestock Equipment, Inglewood, SA Australia), above a solid mat but contained no cover.

The MR was 1,800 mm in width by 2,350 mm in length, with a triangular creep area, measuring 1,070 mm deep and 920 mm wide. A back gate diagonal to the front gate provided two entry points to the pen. The ring was installed on polygrate plastic slatted flooring (Stockyard Industries, North Bendigo, VIC, Australia) and placed on a diagonal within the pen with internal dimensions being 1,160 mm in width and 2,060 mm in length and installed at a height of 250 mm from the floor. The triangular piglet creep area (0.49 m^2^) was heated via mat (RHS120, Stockyard Industries, North Bendigo, VIC, Australia) and fitted with a hinged lid and an LED light designed to attract piglets (29).

### 2.3 Measurements

Production data were collected for the number of piglets born (total, born alive, and born dead) and the number of piglets weaned per sow. At 07:00 the day after farrowing, the face of each sow was inspected and allocated a facial injury score based on the spread of abrasions across three zones of the sows’ head (nose, snout and eyes/ears) as defined by Plush et al. (30). Due to the low incidence of sows presenting with a higher score (>2), this was converted to a binary trait (yes or no for facial injuries). Each sow was measured for body condition using backfat depth at the P2 site (ImaGo.S, IMV imaging, Rochester, MN, United States) on entry and exit to the farrowing house. At weaning, the presence or absence of udder damage (skin breakage) and shoulder sore was noted. Any sow or piglet displaying symptoms of illness (sow: ill thrift, vaginal discharge, mastitis, or shoulder sore; piglet: scour, meningitis, ill thrift, and physical injury) were medicated using the farms approved veterinary medication list (generally an antibiotic and non-steroidal anti-inflammatory), and these were recorded. Daily feed intake was measured for a subset of 114 sows (FC *n* = 58; MR *n* = 56). Sows were fed two times daily using a feed cart converted to scales. Each feeder was filled to a standardised volume (marked by a lip at the top of the hopper), and so the feed delivered on each day was summed to obtain the daily feed intake for each sow. Piglets were ear-tagged for individual identification, and litter size and weight were recorded after cross-fostering at 24 h and again at 18 days of age. Piglets were classified as having failed weaning if they weighed less than 3.5 kg at this time. Lactation efficiency was calculated by dividing the total feed intake by the litter weight weaned at day 18.

### 2.4 Behavioural analysis

The following behavioural measures were analysed using footage recorded using cameras (GoPro, San Mateo, California, United States) mounted overhead farrowing accommodation. Seventeen sows (parity 2–4) were selected from each treatment; however, issues with field of view, recording duration, and image quality resulted in some footage being disregarded. The resultant sample size for each of the behavioural tests is outlined in Table 1. A single observer scored each of the behavioural tests.

**Table 1:** Sample size (*n*) for each of the behavioural tests analysed for sows housed in a farrowing crate (FC) or Maternity Ring (MR).

	FC	MR
*n sows for analyses*		
Anticipatory behaviour (days-2, 12, and 18)	17	17
Startle test (days-2 and 18)	9	12
Farrowing (day 0)	11	15
Sow separation test (day 2)	17	14
Time budgets (days 5 and 20)	13	12

#### 2.4.1 Anticipatory behaviour

On days −2, 12, and 18 relative to farrowing, the anticipatory behaviours of 17 sows per treatment were analysed prior to a feeding event using continuous sampling. Sow behaviour was recorded for 10 min prior to the test being conducted (termed ‘pre-test’). Anticipatory behaviours were elicited by pushing a feed cart through the shed and waiting for 3 min before commencing feed delivery (termed ‘during test’). The duration of time spent performing each behaviour (defined in Table 2) was scored during both the ‘pre-test’ and ‘during test’ periods. As the time taken for feed delivery (and so ‘during the test’ period) differed for each sow based on location within the shed, data were converted to the proportion of time spent performing each behaviour.

**Table 2:** Ethogram of the behaviours recorded during the anticipatory test [adapted from Doyle et al. (27)].

Behaviour	Definition
Lying	Sow is lying down ventrally or laterally
Sitting	Hindquarters on the ground, sternum elevated, head between two top bars
Standing	On all four feet, head between two overhead bars/in the centre of the crate
Head movements around the bars	Snout is above the feeder or between/above side rails surrounding the feeder and includes when lying down or suckling
Feeder interaction	Rubbing snout on feeder, biting feeder, pawing/climbing feeder
Frustrated behaviours	Head shake, stepping without changing location, bar biting, pawing, climbing, nosing repeatedly away from feeder
Drinking	Snout on nipple drinker
Urinate/defecate	Sow excretes urine or faeces
Suckling	Lying laterally with >70% of piglets at the udder

#### 2.4.2 Startle test

The startle response of 9 FC and 12 MR sows to a loud, unexpected stimulus, a metal pipe hitting a solid metal gate placed in the middle of the test zone, was recorded on days −2 and 18 relative to farrowing. On each test day, the sound stimulus was played three times (labelled startle 1–3) at 3-min intervals. The magnitude of the startle response displayed by each sow was scored using the 6-point scale developed by Doyle et al. (27) with 0, no response; 1, ears move; 2, head moves and no freeze; 3, head moves and short freeze (≤5 s); 4, head moves and long freeze (>5 s); 5, flinch and freeze (body changes position).

#### 2.4.3 Farrowing

Farrowing behaviours (Table 3) were recorded for 11 FC and 15 MR sows from the birth of the first piglet until placenta expulsion and scored continuously. In addition to the farrowing duration and the mean piglet birth interval, the minimum, maximum, standard deviation, and coefficient of variance piglet birth interval were calculated for each sow.

**Table 3:** Ethogram of behaviours recorded during farrowing.

Behaviour	Definition
*Pain-related/frustrated behaviour*	
Posture change	Sow moves between standing, sitting, lying lateral, and lying ventral
Bar biting	Biting bars with mouth
Back leg forward	Lateral lying position, the back leg is pulled forward and/or in towards the body
Straining	Lateral lying position, legs lift and push away, straining by muscle clenching
Back arch	In lateral lying position, sow arches back in a concaved manner

#### 2.4.4 Time budget

Sow behaviour was analysed for 13 FC and 12 MR sows on days 5 and 20 relative to farrowing between 10:00 and 14:00. Behaviour was scored using scan sampling with a 30-s interval according to Table 4.

**Table 4:** Ethogram for maternal behaviours at days 5 and 20 of lactation.

Behaviour	Definition
*Posture*	
Sitting	Hindquarters on the ground, sternum elevated
Standing	Sow is standing upright with all feet on the floor
Lying ventrally	Sow lies on udder
Lying laterally	Sow lies on the side
*Activity*	
Eating	Sow pulls feed from feeder into mouth and chews
Drinking	Sow activates water nipple with mouth or nose
Stereotypies	Nosing crate fixtures repeatedly in a fixed manner over a short period of time, champing involving repetitious opening and closing of the mouth resulting in saliva foam, and biting of any bar within the pen
Piglet interaction	Sow noses or nuzzles piglet, or the piglet approaches sows face and nuzzles or nudges it
Active/Alert	Sow is awake but not engaged in the other defined behaviours. May be alert in any posture
Resting/inactive	Sow is asleep/resting and is not inquisitive or looking around at her environment
Nursing	70% of the litter is suckling; sow appears to be grunting and letting down, maybe in standing, sitting, or lying posture
*Position*	
1 to 12	Position of sow based on numbers on an analogue clock face; position 12 oriented with the head directly facing the feeder (31)

#### 2.4.5 Sow separation

Piglets from 17 FC and 14 MR were separated from the sow at 2 days of age for the purpose of piglet processing. The time taken to process each litter was recorded, in addition to the time taken from the first piglet removal until the initial sow reaction. The strength of the sow reaction was scored according to Doyle et al. (27) with 0: no reaction; 1, weak reaction, head movement towards piglet being removed; 2, medium reaction, body movement towards piglet being removed; and 3, strong reaction, attack towards experimenter removing piglet. Once all piglets were placed back with the sow, the footage was analysed for a further 5 min for behaviours as described in Table 5. Rather than a number of events, the incidence of sows that performed eating/drinking, frustrated and pre-let-down behaviours were examined.

**Table 5:** Ethogram of the behaviours recorded during the separation test.

Behaviour	Description of behaviour
*First piglet removal to piglet reintroduction*	
Frustrated behaviours	Bar biting, champing, nosing ground, or hitting/biting feeder
*Post piglet reintroduction to 5 min*	
Positive piglet interaction	Nosing or nuzzling between sow and piglets, initiated by either sow or piglet
Eating or drinking	Sow eats from the feeder or drinks from the nipple
Pre-let-down event	Piglets approach the udder, the sow grunts, exposes the udder and < 70% of piglets start teat seeking or massaging, initiated by either the sow or piglet

### 2.5 Statistical analysis

Data were analysed (IBM SPSS Statistics for Windows, Version 28.0, Armonk, NY, United States) with a *p*-value < 0.05 considered significant, and < 0.10 a tendency. Production, performance, and behavioural data were analysed using negative binomial regression (count data), general linear mixed models (continuous data), and binary regression (yes/no data) with treatment (FC and MR) as a fixed effect and replicate (1 or 2) as a random term. Non-normally distributed data were transformed, with transformed data presented and back-transformed data in brackets. Farrowing behaviour data that were not normally distributed included birth interval mean and minimum, which were log_10_ transformed, and the amount of time spent sitting, which was square root transformed. Data from the separation test that were not normally distributed included the duration of litter processing and the duration of the first posture change, both of which were log_10_ transformed. Data for the anticipatory test and sow orientation on days 5 and 20 were analysed as the proportion of time a sow spent performing the behaviour, and no transformation normalised the data. Thus, these data were analysed using the non-parametric Kruskal–Wallis test. Data presented are mean ± standard error of the mean (SEM).

## 3 Results

During farrowing, there was no difference in the total farrowing duration (260.6 ± 27.76 min) or piglet birthing intervals (*p* > 0.05; Table 6). FC sows performed bar biting more frequently than MR sows (*p* = 0.038). There were no differences in posture changes, straining or back arching (p > 0.05), but the number of times a sow moved her back leg forward was increased in FC sows (*p* = 0.019).

**Table 6:** Mean ± SEM farrowing performance and frequency of behaviours for sows housed in a farrowing crate (FC) or Maternity Ring (MR).

	FC		MR		
	Mean	SEM	Mean	SEM	*p*-value
*Number of events*					
Posture change	32.1	9.97	26.1	6.95	0.62
Bar biting	3.3	2.12	0.5	0.29	0.038
Back leg forward	227.3	50.69	126.5	26.44	0.019
Straining	221.5	53.00	206.1	46.59	0.76
Back arch	6.7	9.66	11.4	15.64	0.70
*Piglet birth interval*					
Mean (min)^*^	3.1	0.08	3.1	0.06	0.65
	(21.1)		(19.1)		
Minimum (min)^*^	1.4	0.25	1.5	0.2	0.77
	(0.4)		(0.5)		
Maximum (min)	95.2	18.41	73.3	15.42	0.35
Standard deviation	31.6	7.27	21.8	5.94	0.31
Coefficient of variation (%)	1.8	0.11	1.7	0.09	0.55

^*^Back-transformed data presented in brackets.

There was no difference in mean number of piglets total born (13.6 ± 1.56), born alive (12.8 ± 1.47), born dead (0.8 ± 0.14), and weaned (9.9 ± 1.16) between FC and MR sows (*p* > 0.05). Fewer MR sows presented with facial injuries the day after farrowing (10% CI [5, 20] vs. FC; 67% CI [47, 95] *p* < 0.001), despite both treatments showing no incidence of injury at farrowing house entry. Sow reaction score to piglet processing tended to be higher for MR sows (2.0 ± 0.38 vs. FC 1.2 ± 0.27; *p* = 0.09). The duration of piglet processing was 1 min longer in MR compared to FC (*p* = 0.008), but there was no difference in duration to the first posture change (*p* > 0.05) or number of frustrated behaviours (*p* > 0.05) between the two systems (Table 7). After piglet return, there were more piglet interactions in the MR (*p* = 0.016), driven by increased sow-initiated interactions (*p* = 0.027). More FC sows were observed to eat/drink than MR (*p* = 0.05), but no difference in the incidence of sows that displayed frustrated or let-down behaviours was found (*p* > 0.05).

**Table 7:** Mean ± SEM behaviour during the separation test for sows housed in a farrowing crate (FC) or Maternity Ring (MR).

	FC		MR		
	Mean	SEM	Mean	SEM	*p*-value
*First piglet removal to piglet reintroduction*					
Processing duration (sec)^*^	2.2	0.03	2.4	0.03	0.008
	(204)		(264)		
Duration to posture change (sec)^*^	1.1	0.16	0.9	0.18	0.46
	(11.7)		(7.7)		
Frustrated behaviour (n)	7.1	1.96	6.2	1.91	0.76
Sows displayed frustrated behaviours (%)Ɨ	80	55–95	90	56–99	0.26
*Post piglet reintroduction*					
Positive piglet interactions (n)	6.9	1.26	13.5	2.55	0.016
Piglet initiated positive interactions (n)	2.8	0.73	4.8	1.29	0.16
Sow initiated positive interactions (n)	4.1	0.94	8.8	2.03	0.027
Sows ate or drank (%)Ɨ	50	16–10	10	3–48	0.05
Sows exhibited a pre-let-down event (%)Ɨ	20	5–51	40	17–75	0.16

^*^Back-transformed data presented in brackets, Ɨ95% confidence intervals given for binary traits rather than SEM.

When time budgets were examined, MR sows tended to spend more time sitting on day 5 of lactation when compared to FC sows (*p* = 0.056; Table 8). On day 20 of lactation, MR sows interacted with their piglets significantly more than FC sows (*p* = 0.046). There were no differences in the other recorded maternal behaviours (*p* > 0.05).

**Table 8:** Mean ± SEM of number of times behaviours were observed over a 4-h period scored at 30-s intervals (480 observations in total) on day 5 and day 20 of lactation for sows housed in a farrowing crate (FC) or Maternity Ring (MR).

	Day 5 of lactation					Day 20 of lactation				
	FC		MR			FC		MR		
	Mean	SEM	Mean	SEM	*p*-value	Mean	SEM	Mean	SEM	*p*-value
*Posture*										
Sitting	3	2.5	13	10.2	0.056	6	2.8	14	5.4	0.19
Standing	35	13.5	32	10.3	0.86	66	35.5	57	30.5	0.75
Lying ventrally	64	14.5	77	19.1	0.57	102	25.5	150	34.3	0.26
Lying laterally	281	60.0	339	74.4	0.34	317	70.9	238	52.8	0.12
*Behaviour*										
Eating	38	12.5	37	9.6	0.96	57	14.1	45	10.4	0.34
Drinking	10	2.0	7	1.5	0.19	17	4.3	15	3.6	0.79
Stereotypy	4	1.2	5	1.4	0.58	4	1.5	8	3.6	0.79
Piglet interaction	6	1.5	7	1.7	0.62	3	0.99	7	2.0	0.046
Active	25	4.4	32	5.6	0.38	43	15.3	42	14.9	0.97
Resting	304	50.4	373	63.6	0.19	351	39.8	642	38.5	0.69
Nursing	13	2.4	13	2.6	0.88	11	1.1	12	1.0	0.79

**Figure 2:**
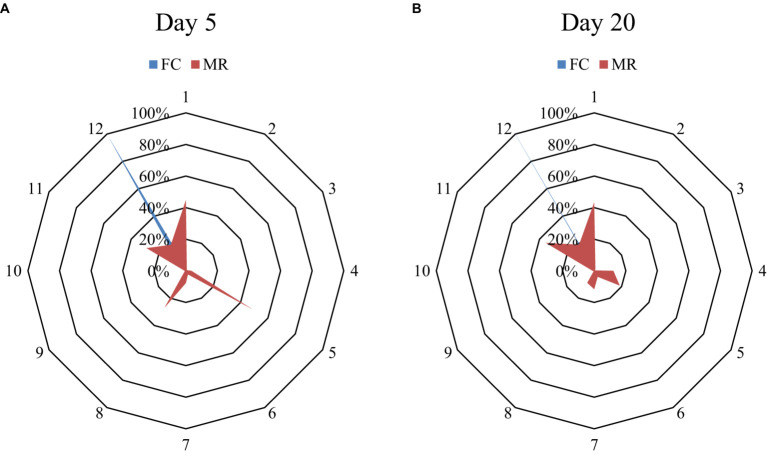
Percent of observations scored at each position over a four-hour period at 30 second intervals (480 observations in total) on day 5 **(A)** and day 20 **(B)** of lactation for sows housed in a farrowing crate (FC) or Maternity Ring (MR). Position 12 represented the feeder and position 6 the opposite corner to the feeder. An increasing distance between the data point representing each position from the center point of each graph indicates an increasing proportion of observations with the head oriented towards this position. The shaded area represents the percent of observations for which sows were oriented in each direction (adapted from (31)).

Orientation of the sow was significantly different at both day 5 and day 20 between the MR and FC systems (*p* < 0.001). Sows in the MR spent less time facing the feeder on both day 5 and day 20 (19.0 ± 6.36 and 19.1 ± 5.79) than FC sows (96.4 ± 43.17 and 99.8 ± 44.69). The amount of time sows spent in each location within the pen (represented by a clockface) is presented in Figure 2.

**Figure 3:**
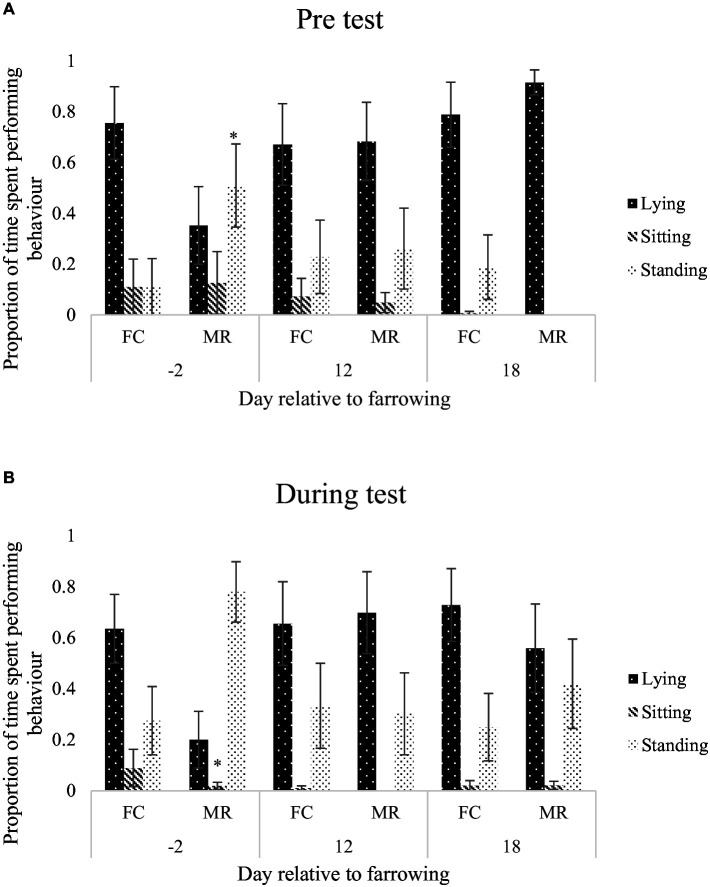
Mean ± SEM proportion of time spent lying, sitting, or standing on day −2, 12, or 18, relative to farrowing, prior to anticipatory test **(A)** and during the anticipatory test **(B)**, when sows were housed in either a farrowing crate (FC) or Maternity Ring (MR). ^*^Represents significant difference in specified behaviour between treatment within day.

**Figure 4:**
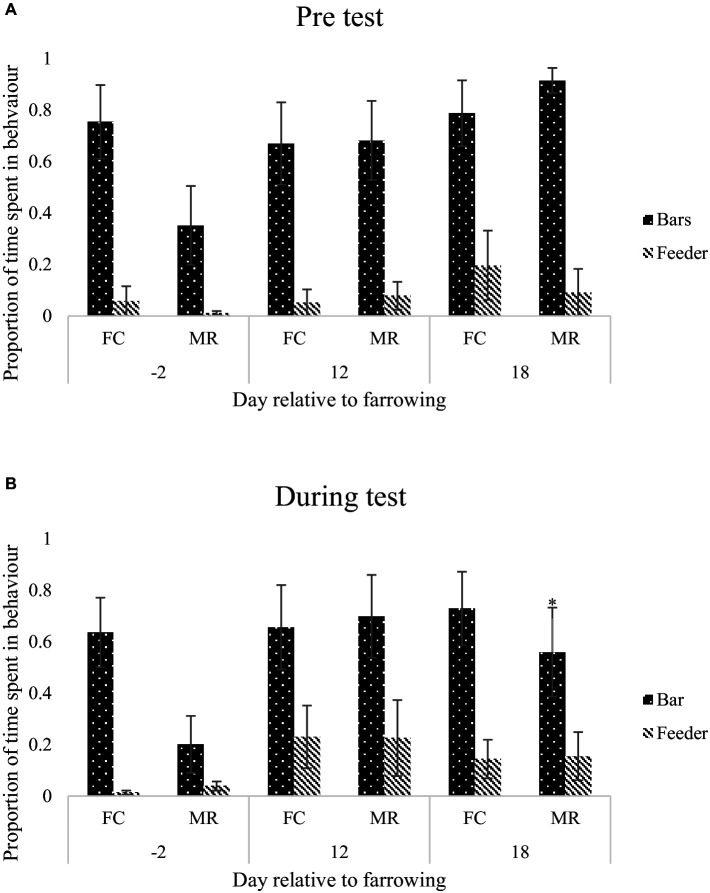
Mean ± SEM proportion of time spent interacting with the bars around the accommodation or feeder on day −2, 12, or 18 relative to farrowing, prior to anticipatory test **(A)** and during the anticipatory test **(B)**, when sows were housed in either a farrowing crate (FC) or Maternity Ring (MR). ^*^Represents significant difference in specified behaviour between treatment within day.

For the anticipatory behaviour test, MR sows spent more time standing during the pre-test period on day −2 than FC sows (*p* = 0.048; Figure 3A). During the test period on day −2, FC sows spent more time sitting than MR sows (*p* = 0.022; Figure 3B). There was no difference in interactions with bars or the feeder during the pre-test period on any day for the anticipation test (*p* > 0.05), but on day 18 of lactation during the test period, FC sows interacted with the bars of the crate more than MR sows (*p* < 0.001; Figures 4A,B). Frustrated behaviours were not different between FC and MR sows on any measurement day (*p* > 0.05).

**Figure 5:**
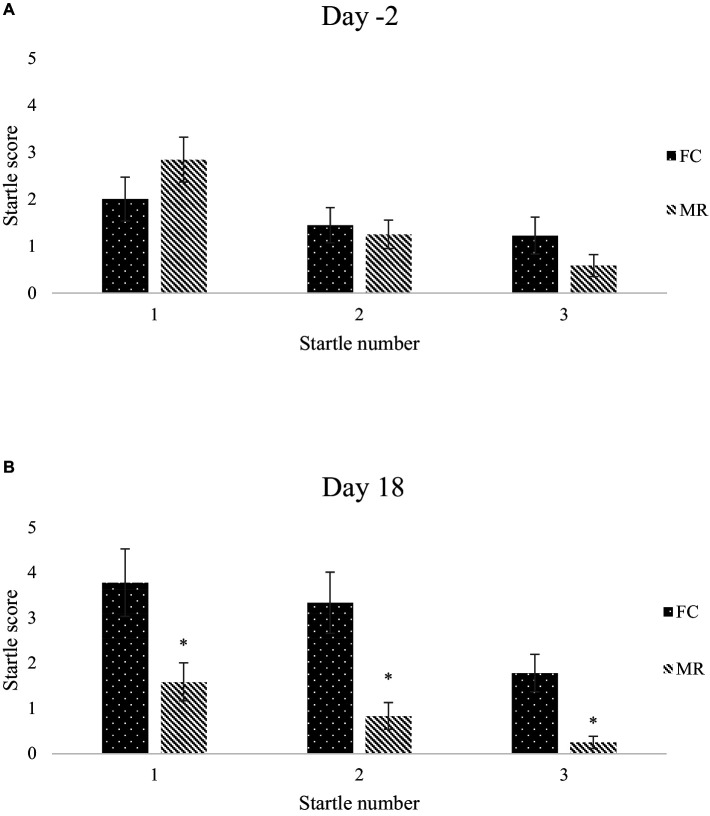
Mean ± SEM startle score recorded after aversive stimulus imposed three times at 3-min intervals on day −2 **(A)** and day 18 **(B)** relative to farrowing when sows were housed in either a farrowing crate (FC) or Maternity Ring (MR). ^*^Represents significant difference between treatment within test.

Prior to farrowing (day-2), there was no difference in startle score; however, by day 18 FC sows received a higher startle score at every event when compared with MR sows (Figure 5).

On days 1 and 18 of lactation, litter sizes in FC and MR were similar. Litter weight tended to be heavier on day 1 (*p* = 0.083) and became significantly heavier by day 18 (*p* = 0.049) in the FC treatment. Average individual piglet weight was not different on day 1 (*p* > 0.05), but by day 18, piglets reared in MR were heavier (*p* = 0.004). More piglets reared in FC failed the weaning event (*p* = 0.008). There was a tendency for more MR sows to require medical intervention than FC sows (*p* = 0.054). Litters reared in FC required medical intervention more frequently than piglets reared in MR (p = 0.008). There was no difference in the presence of shoulder sores (*p* > 0.05); however, at weaning more FC sows recorded udder damage than MR sows (*p* < 0.001; Table 9).

**Table 9:** Mean ± SEM of measures of sow and litter performance and health in farrowing crates (FC) or Maternity Rings (MR).

	FC		MR		
	Mean	SEM	Mean	SEM	*p*-value
Litter size day 1	12.1	1.40	12.1	1.41	0.98
Litter size day 18	10.6	1.24	9.3	1.09	0.41
Litter weight day 1 (kg)	16.7	0.33	15.9	0.33	0.083
Litter weight day 18 (kg)	49.6	1.17	46.4	1.17	0.046
Average piglet weight day 1 (kg)	1.38	0.027	1.33	0.027	0.13
Average piglet weight day 18 (kg)	4.68	0.080	5.00	0.080	0.004
Number of piglets < 3.5 kg on day 18	2.2	0.55	0.9	0.25	0.008
Sows medicated (%)^*^	4	1–11	12	7–21	0.054
Litters medicated (%)^*^	51	40–62	30	21–41	0.008
Sows with shoulder sores (%)^*^	16	8–28	15	8–28	0.85
Sows with udder damage (%)^*^	69	48–9	12	6–24	<0.001
Entry P2 backfat (mm)	18.8	0.13	18.4	0.13	0.043
Exit P2 backfat (mm)	17.6	0.10	18.4	0.09	<0.001
Total feed intake (TFI) to D18 (kg)	111.2	3.13	93.8	3.06	<0.001
Lactation efficiency to D18 (kg TFI/ kg litter weight D18)	2.3	0.08	2.1	0.08	0.062
Wean to first service interval (days)	9.0	1.13	6.7	0.82	0.088

^*^95% CI presented rather than SEM presented for binary data.

FC sows recorded a higher P2 backfat depth at entry (*p* = 0.043) but were lower at exit (p < 0.001), whereas sows housed in the MR remained the same throughout lactation. FC sows had higher total feed intake from farrowing until day 18 (*p* < 0.001). Sows from the MR treatment tended to be more efficient, recording 200 g less feed to produce 1 kg of litter weight weaned (*p* = 0.062), as well as a tendency for reduced wean to first service interval (*p* = 0.088; Table 9).

## 4 Discussion

The aim of this experiment was to assess the welfare of sows at farrowing and during lactation housed within a farrowing crate in comparison to a free farrowing alternative with a similar footprint, the Maternity Ring, using the Five Domains framework. Results indicated that sows housed in the MR had improved lactation efficiency, more ability to observe and interact with the environment both within and outside the pen, displayed reduced injuries, performed more species-specific behaviours such as nesting and piglet bonding, and exhibited an improved response to a startling test. This all contributes to a substantial improvement in sow welfare indicating a more positive affect when compared to sows in farrowing crates. Our hypothesis is supported, with sows housed in the MR having superior welfare to those housed in farrowing crates.

### 4.1 Nutrition

We had expected that sows housed in the MR treatment would show increased feed intake as has been reported previously (32), due to a greater freedom of movement leading to ease of standing and eating, and higher basal metabolic requirements. We did not find evidence of this, and in fact, crated sows were delivered significantly more feed over lactation. However, lactation efficiency tended to be higher in MR than in FC sows. Although not measured, the crated sows may have experienced increased chronic stress (33) and, as the intensity of the adrenal response to ACTH in pigs is negatively related to feed efficiency and growth rate (34, 35), acted to reduce lactation efficiency. This has sustainability outcomes other than animal welfare as if the kilogrammes of pig meat produced per sow feed consumed is improved in the Maternity Ring, the environmental footprint of the system is reduced (13).

Despite the reduced feed intake, MR sows were better able to maintain body condition than sows in crates, as measured by the change in P2 backfat thickness. FC sows lost more than 1 millimetre of backfat throughout lactation, whereas MR sows maintained condition to weaning. This maintenance of the P2 backfat may help to explain why there was a tendency for a reduced wean to the first service interval. The effect of sow body tissue mobilisation on subsequent reproduction is well documented (36–38).

Despite efforts made to reduce feed wastage, we did observe that crated sows appeared to waste more feed than those in the MR. Nine crated sows were removed from measures of feed intake due to obvious signs of excessive wastage (visible feed accruing under the slatted flooring), whilst none were removed from MR treatment (data not presented). Feeder interactions that did not involve actual consumption were measured and classified as stereotypic behaviour; however, there was no difference in stereotypies between the two treatments in the time budget analyses. Further study should be conducted into the specific feeding behaviours of crated vs. penned sows, as crated sows have no ability to turn away from the feeder, which may lead to eating for stereotypic reasons and not nutritional needs. As it is generally accepted that feed is the largest cost to pig farming enterprises, any reduction in wastage would have substantial financial benefits.

### 4.2 Environment

The sow’s choice of orientation is an important factor in assessing welfare, as hindered and/or enhanced expression of agency affects animals’ ability to interact with their environment (17). The MR sows were able to make a conscious choice in orientation (i.e., turn around unhindered) and so were able to actively engage with the physical and social environment beyond the degree demanded by their momentary needs. This allowed the sow to gather knowledge and enhance skills for future use in responding effectively to varied and novel challenges (39–41). The MR sows spent an average of 75% of their time facing away from the feeder, whereas in the FC treatment, this represented only 2% of observations during the time budget assessment. Therefore, when given the option a sow will make the conscious choice to spend a significant amount of time facing away from the feeder, an option not available to crated sows.

There was a reduced number of MR sows presenting with facial injuries at farrowing and so presumably less nesting behaviours were directed at hard fixtures, rather than FC sows. When confined to a barren environment, sows will perform nesting behaviours by nosing and pawing crate fixtures, becoming stereotypic in nature and resulting in injuries that can be observed on the face (42). Crated sows in this experiment were provided with hessian and so did not farrow in a barren environment; however, the number of bar biting events during farrowing and the rate of facial injury was still significantly higher. Therefore, it is the greater freedom of movement in combination with a nesting substrate that allows the sow to satisfy the need to nest build.

### 4.3 Health

Almost 70% of crated sows were recorded as having udder damage at weaning, in comparison with only 12% of MR sows. Previous studies have associated increased skin lesions on the udder with lying positions (43) and flooring types (44). In the present experiment, there were no differences in lying behaviours observed during the time budget assessment, and both systems were equipped with identical plastic slatted flooring. Pedersen et al. (45) observed more teat fights between piglets and more restlessness during suckling in crated systems, and whilst teat disputes were not analysed in this experiment, this is the most likely explanation for reduced udder injuries in MR sows. Anecdotally, sows housed in the MR were able to stretch out front and back legs, allowing greater space at the udder for milk let-down events as lactation progressed and piglets grew. The position of sows during milk let-down events, and piglet disputes during feeding need to be quantified in the two systems to confirm this suggestion.

Crating sows, thereby limiting movement, has been shown to increase the incidence of ailments such as lameness and other disorders (46) (1) that require medical intervention, and so it was expected that the occurrence of such events would be lower in MR sows. We identified a trend for increased medical intervention in the MR sows in opposition to this sentiment. Six out of the 10 sows treated in the MR group came into farrowing accommodation with pre-existing ailments from gestation (one out of three in FC sows), and so the effect on medical interventions was not likely caused by the housing treatments.

Interestingly, piglet medications were fewer in the MR group. When the reasons for medical intervention were examined, the farm staff recorded more litters were treated for ‘meningitis’ in crated litters, responsible for 35% of all treatments, in comparison with 12% in the MR piglets. Meningitis is caused by *Streptococcus suis*, which lies dormant in the tonsils of a piglet often causing meningitis secondary to another condition, which in the pre-weaning period is most likely due to additional disease challenge or stress (47). As previously discussed, the increased udder damage observed on the FC sows may represent higher levels of teat disputes during feeding events, which could be interpreted as a stressful event for piglets and so the likelihood of meningitis symptoms. Alternatively, Nowland et al. (48) identified that piglets born to free farrowing sows ingested more colostrum which may have improved immunological status as so protection against disease. Future study should examine the welfare of piglets reared in the Maternity Ring.

There was no difference in litter size at day 1 or 18 of lactation, although numerically FC sows recorded 1.5 extra pigs at day 18. This did not translate to more pigs weaned, however, as the experimental site excluded pigs from entering the nursery facility when weighing less than 3.5 kg, with this failure rate being 1.3 pigs per litter higher in the FC sows. Piglet medications were 21% higher in crated litters which may explain this finding. The pigs that failed to wean were not removed from the system but placed on nurse sows for more lactation days to reach minimum weaning weight requirements, translating to additional labour as well as lost productive sow days and crate spaces.

### 4.4 Behaviour

The number of times a sow moved her back leg forward and performed bar biting was higher in FC sows, behaviours thought to indicate a more painful farrowing event (49). These findings are in support of those by Nowland et al. (48), who concluded that the reduced level of perceived pain may be explained by the ability of non-crated sows to move freely and become comfortable, or changes in endocrinology that results in parturition-induced hypoalgesia. Previous studies have found the level of confinement experienced by sows around farrowing to have significant physiological effects on the level of cortisol prior to farrowing (50) and the duration of farrowing (51). However, in this experiment, there were no observed differences in farrowing duration or piglet birth interval. This result is surprising as the increase in facial injury score, bar biting events, and tendency for increased stereotypical behaviours in FC sows would generally be considered indicative of increased stress around farrowing (42, 50, 52, 53). Whilst no changes in farrowing duration or intrapartum piglet deaths were observed, the behavioural observations indicate that MR sows were experiencing a less stressful farrowing process when compared to FC sows.

In early and late lactation, there was little indication of behavioural divergence between the two housing systems, which contrasts previous study that have identified shifts in both activity levels and nursing behaviour in free movement sows (54–56). This may be due to the reduced spatial footprint of the MR in comparison with other pen designs, or alternatively, the environmental conditions experienced. The experiment was conducted in naturally ventilated sheds during summer with high ambient temperatures, and observations took place during the warmest part of the day (10:00–14:00), which may reduce behavioural divergence in place of thermoregulatory comfort. In support of this, in approximately 75% of observations, the sows were scored as resting/inactive. The observation period was selected to limit the amount of disturbance during routine husbandry procedures and so stockperson interference; however, future research may benefit from investigation of sow behaviour around the busier periods in the morning and afternoon, when it is not only cooler but also when sows are more alert, active, and have more interaction with farm staff.

Sows in the MR showed a greater response to the removal of piglets during litter processing and this is not dissimilar to previous findings (57). Grimberg-Henrici et al. (58) showed that sows in confinement-free farrowing pens showed improved maternal behaviour more responsive to distress calls from their piglets. It has been suggested that the ability to perform a nose-to-nose interaction between the sow and her litter is essential in facilitating mother–offspring bonding (59, 60). Thus, the observed increase in positive interactions with piglets in the MR facilitated a stronger maternal bond and so greater reactivity when piglets were removed for processing. In support of this, the proportion of sows that performed maintenance behaviour such as eating or drinking during separation from their piglets was higher in the FC sows, and these sows had fewer contact events with piglets upon their return, indicating that crated sows were less reactive to piglet separation. This was sustained through to later lactation, where once again when observing the litters undisturbed, MR sows had more than double the contact with piglets.

### 4.5 Mental state

The Five Domains model accepts that improved nutrition, environment, health, and behavioural conditions result in a shift in mental state of the animal towards a more positive affect (16). Mother–infant bonding is an intrinsic maternally driven process, beginning with essential nose-to-nose interactions during farrowing in the pig (Gunlach et al., 1968) (59, 60). External circumstances that enable social species to engage fully in bonding, allogrooming, play, and sexual activities have been suggested to give rise to positive affects, contributing to enhanced welfare (17). Affects that are potentially associated with aspects of bond affirmation and maternal care are of particular interest and include feeling engaged, affectionately sociable, and maternally rewarded (61–63). We suggest that MR sows experienced a more positive affect due to the increased level of interaction with piglets that was reported both during and after the sow separation test conducted during early lactation and time budget assessment occurring in late lactation.

Variation in defence cascade response is a potential indicator of affective valence and hence welfare in pigs (23). Two startle tests were conducted whilst the sows were in the farrowing accommodation. As expected, the first startle test revealed no differences in behavioural response, as the sows had not been in the accommodation long enough to alter their welfare state, therefore acting as a baseline measurement. In later lactation, sows housed in the MR displayed a lower startle score at every test, and by the final test, the score was almost 0 whilst those in an FC still recorded a score of almost two. The ability of the MR sows to turn around and investigate the environment throughout lactation possibly allowed them to gather knowledge and enhance their skills for future use in responding effectively to varied and novel stimuli (39–41). Anecdotally, it was observed that after the first aversive noise of the test, MR sows would startle and turn to investigate the source. The ability to turn allowed these sows to investigate the environment for the perceived threat, after which they would return to their normal behaviour and show attenuated responses to the remaining two aversive noises. Maternity Ring sows have a better control (and possibly agency) in their environment (as being able to assess a potential threat) and thus do not have an exacerbated response to a startling stimulus.

In the current experiment, a feed cart was presented to MR and FC sows without feed delivery with the hope of eliciting increased anticipatory behavioural patterns. Feed delivery, when restricted feeding is applied, will elicit anticipatory behaviour due to the biological needs of the sow (64). However, in the current experiment sows were fed at high levels to induce satiety prior to farrowing and *ad libitum* during lactation and so were not restrict-fed. If the sows were restrict-fed, this may have had adverse effects both with regard to the affect of the sows, and the impact on other recorded measures. Future study should involve the training of sows to receive a reward signalled with a cue, with anticipation measured in response to this delivery. This would have constituted a positive event, unrelated to unmet biological needs.

## 5 Conclusion

This experiment aimed to assess the welfare of sows housed within a farrowing crate in comparison with a free farrowing alternative with a similar space requirement, the Maternity Ring, using the Five Domains framework. We were able to successfully demonstrate improvements in sow nutrition, environment, health, and behaviour that all contributed to a positive shift in mental state. Thus, our hypothesis is supported, and we can conclude that sows housed in the Maternity Ring experience higher animal welfare standards than those housed in farrowing crates. The Maternity Ring can improve sow welfare whilst preserving sow space in farrowing units.

## Data availability statement

The raw data supporting the conclusions of this article will be made available by the authors, without undue reservation.

## Ethics statement

The animal study was approved by Primary Industries and Resources South Australia Animal Ethics Committee. The study was conducted in accordance with the local legislation and institutional requirements.

## Author contributions

KP: Conceptualization, Formal analysis, Methodology, Project administration, Supervision, Writing – original draft, Writing – review & editing. DL: Conceptualization, Writing – review & editing. LS: Conceptualization, Data curation, Formal analysis, Investigation, Methodology, Writing – original draft, Writing – review & editing. DD: Conceptualization, Supervision, Writing – review & editing. RvB: Conceptualization, Supervision, Writing – review & editing.
