# Supplementary material for: A five domains assessment of sow welfare in a novel free farrowing system
Source: Front Vet Sci. 2024 Aug 12;11:1339947. doi: 10.3389/fvets.2024.1339947 (PMC11370643; doi:10.3389/fvets.2024.1339947)
Supplement: Supplementary file 1 [file Data_Sheet_1.zip › Supplementary Material Presentation/Supplementary_Material - figure 1.docx]

Supplementary Material

## Supplementary Figures


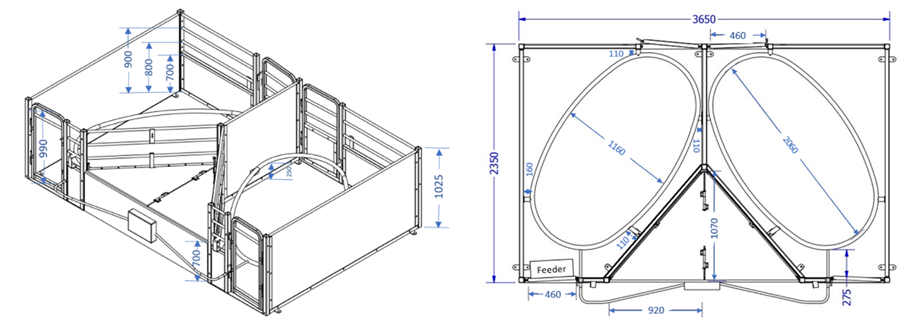


**Figure 1.** Dimensions of key design features of the Maternity Ring.
