# Supplementary material for: A five domains assessment of sow welfare in a novel free farrowing system
Source: Front Vet Sci. 2024 Aug 12;11:1339947. doi: 10.3389/fvets.2024.1339947 (PMC11370643; doi:10.3389/fvets.2024.1339947)
Supplement: Supplementary file 1 [file Data_Sheet_1.zip › Supplementary Material Presentation/Supplementary_Material - figure 2.docx]

Supplementary Material

## Supplementary Figures

**Figure 2.** Percent of observations scored at each position over a four-hour period at 30 second intervals (480 observations in total) on day 5 (A) and day 20 (B) of lactation for sows housed in a farrowing crate (FC) or Maternity Ring (MR). Position 12 represented the feeder and position 6 the opposite corner to the feeder. An increasing distance between the data point representing each position from the center point of each graph indicates an increasing proportion of observations with the head oriented towards this position. The shaded area represents the percent of observations for which sows were oriented in each direction (adapted from Kinane *et al.,* 2022).
