# Supplementary material for: A five domains assessment of sow welfare in a novel free farrowing system
Source: Front Vet Sci. 2024 Aug 12;11:1339947. doi: 10.3389/fvets.2024.1339947 (PMC11370643; doi:10.3389/fvets.2024.1339947)
Supplement: Supplementary file 1 [file Data_Sheet_1.zip › Supplementary Material Presentation/Supplementary_Material - figure 4.docx]

Supplementary Material

## Supplementary Figures

**Figure 4.** Mean ± SEM proportion of time spent interacting with the bars around the accommodation or feeder on day –2, 12 or 18 relative to farrowing, prior to anticipatory test (A) and during the anticipatory test (B), when sows were housed in either a Farrowing Crate (FC) or Maternity Ring (MR). *represents significant difference in specified behaviour between treatment within day.
