# Supplementary material for: A five domains assessment of sow welfare in a novel free farrowing system
Source: Front Vet Sci. 2024 Aug 12;11:1339947. doi: 10.3389/fvets.2024.1339947 (PMC11370643; doi:10.3389/fvets.2024.1339947)
Supplement: Supplementary file 1 [file Data_Sheet_1.zip › Supplementary Material Presentation/Supplementary_Material - figure 5.docx]

Supplementary Material

## Supplementary Figures

**Figure 5.** Mean ± SEM startle score recorded after aversive stimulus imposed three times at three-minute intervals on day –2 (A) and day 18 (B) relative to farrowing when sows were housed in either a Farrowing Crate (FC) or Maternity Ring (MR). *represents significant difference between treatment within test.
